# Molecular targeting of protein arginine deiminases to suppress colitis and prevent colon cancer

**DOI:** 10.18632/oncotarget.5937

**Published:** 2015-09-30

**Authors:** Erin E. Witalison, Xiangli Cui, Corey P. Causey, Paul R. Thompson, Lorne J. Hofseth

**Affiliations:** ^1^ Department of Drug Discovery and Biomedical Sciences, South Carolina College of Pharmacy, University of South Carolina, Columbia, SC, USA; ^2^ Shanxi Medical University, Taiyun, China; ^3^ Department of Chemistry, University of North Florida, Jacksonville, FL, USA; ^4^ Department of Biochemistry and Molecular Pharmacology, University of Massachusetts Medical School, Worcester, MA, USA

**Keywords:** protein arginine deiminases, colorectal cancer, cancer prevention, microRNA, epigenetics

## Abstract

Ulcerative colitis (UC) is a chronic disease, in which the lining of the colon becomes inflamed and develops ulcers leading to abdominal pain, diarrhea, and rectal bleeding. The extent of these symptoms depends on disease severity. The protein arginine deiminase (PAD) family of enzymes converts peptidyl-Arginine to peptidyl-Citrulline through citrullination. PADs are dysregulated, with abnormal citrullination in many diseases, including UC and colorectal cancer (CRC). We have developed the small molecule, pan-PAD inhibitor, Chlor-amidine (Cl-amidine), with multiple goals, including treating UC and preventing CRC. Building off our recent results showing that: 1) Cl-amidine suppresses colitis *in vivo* in a dextran sulfate sodium (DSS) mouse model; and 2) Cl-amidine induces microRNA (miR)-16 *in vitro* causing cell cycle arrest, we tested the hypothesis that Cl-amidine can prevent tumorigenesis and that miR-16 induction, by Cl-amidine, may be involved *in vivo*. Consistent with our hypothesis, we present evidence that Cl-amidine, delivered in the drinking water, prevents colon tumorigenesis in our mouse model of colitis-associated CRC where mice are given carcinogenic azoxymethane (AOM), followed by multiple cycles of 2% DSS to induce colitis. To begin identifying mechanisms, we examined the effects of Cl-amidine on miR-16. Results show miR-16 suppression during the colitis-to-cancer sequence in colon epithelial cells, which was rescued by drinking Cl-amidine. Likewise, Ki67 and cellular proliferation targets of miR-16 (Cyclins D1 and E1) were suppressed by Cl-amidine. The decrease in cell proliferation markers and increase in tumor suppressor miRNA expression potentially define a mechanism of how Cl-amidine is suppressing tumorigenesis *in vivo*.

## INTRODUCTION

Ulcerative colitis (UC) is a chronic, relapsing inflammatory bowel disease that affects millions of people worldwide and causes symptoms of abdominal pain, diarrhea, and rectal bleeding. Due to the chronic inflammatory state of UC, which causes cyclical oxidative stress and leads to DNA damage, patients with UC are at a higher risk of developing colorectal cancer (CRC) [1]. The probability of developing CRC increases yearly once UC develops, with up to an approximate 20% incidence after 30 years [1, 2]. Current treatment options help to treat the symptoms, prevent flares, and heal the damaged colon; however, the treatment outcomes are often marginal, patients become refractory, and there are dangerous adverse side effects. Therefore, we continue to investigate novel therapies showing reduced toxicity and more substantial efficacy in treating UC and preventing CRC.

The protein arginine deiminase (PAD) family of enzymes converts peptidyl-Arginine to peptidyl-Citrulline through a process called ‘citrullination’ [3]. There are five isozymes found in mammals (PAD1-4 and 6), with 70-95% homology in their amino acid sequence [4]. The PAD isozymes are encoded by a cluster of homologous genes found on Chromosome 1 in humans [4, 5]. Citrullination can affect protein-protein interactions, hydrogen bond formation, and protein structure due to the shift from the positively charged peptidyl-Arginine to the neutral peptidyl-Citrulline [5]. At physiological activity levels, PADs regulate many cell signaling pathways including differentiation, apoptosis, and gene transcription [6]. However, over the past decade, it is becoming increasingly apparent that aberrant PAD activity is involved in many human inflammatory diseases such as: rheumatoid arthritis, Alzheimer's disease, and multiple sclerosis [3, 7-10]. Concerning our study, PADs, especially PAD4, are found to be dysregulated in UC and CRC [11-14]. In UC and other chronic inflammatory, autoimmune diseases, it is thought that the accumulation of citrullinated proteins is what leads to an abnormal immune response that further exacerbates the inflammatory response [6].

We have developed a novel, small molecule inhibitor of PADs, named N-α-benzoyl-N5-(2-chloro-1-iminoethyl)-L-ornithine amide or Chlor-amidine (Cl-amidine). Cl-amidine prevents citrullination by covalently modifying a conserved cysteine residue in the active site of the PADs, causing irreversible inactivation of the enzyme [6]. Cl-amidine is an excellent candidate drug for our CRC prevention studies because it: 1) exhibits little toxicity at doses we use here *in vitro* and *in vivo*; 2) displays no immunosuppressive effects in multiple disease models associated with PAD dysregulation; 3) effectively suppresses colitis in mice when given by oral gavage; and 4) increases tumor suppressor microRNA (miRNA) levels *in vitro* [14-17]. Based on these precedents, we tested the hypothesis that Cl-amidine can prevent tumorigenesis in the azoxymethane (AOM)/dextran sulfate sodium (DSS) mouse model of colitis-associated CRC. Mechanistic insight is also gained by examining the levels of miRNA changes during this process.

## RESULTS

### Cl-amidine suppresses AOM/DSS-induced tumorigenesis in mice

**Figure 1:**
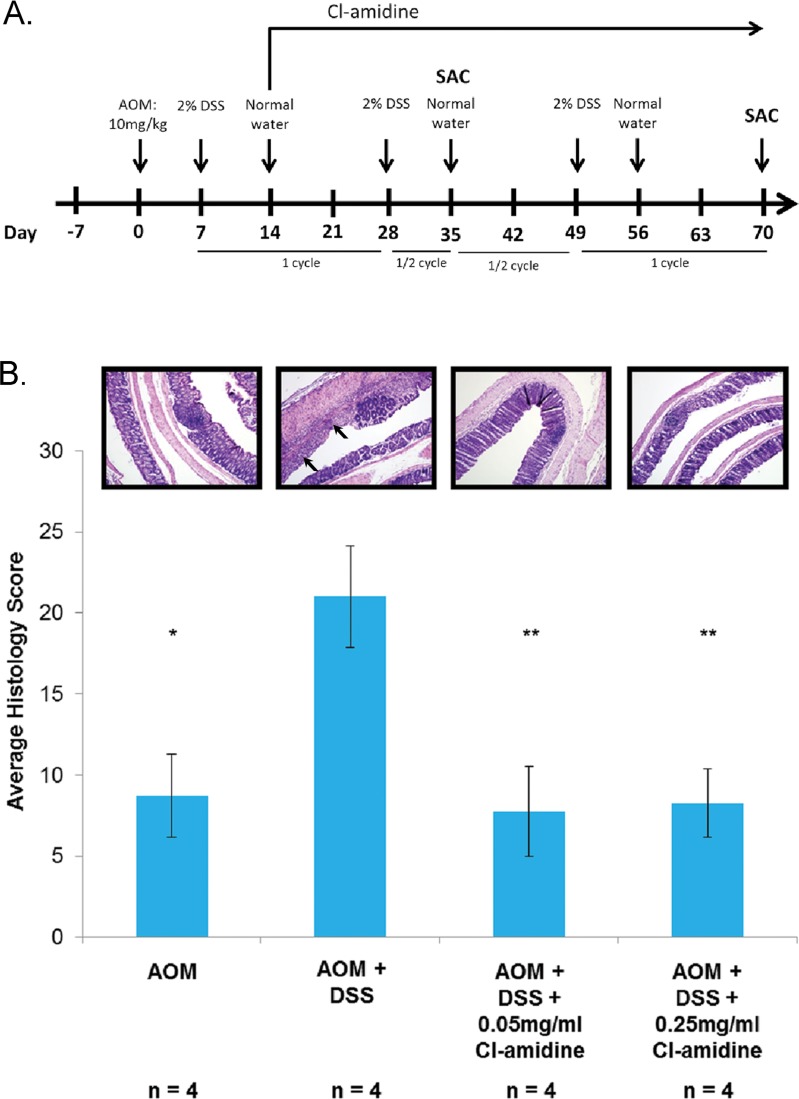
**A.** Outline of the AOM/DSS mouse model of colorectal cancer used in this study. **B.** Histology scoring of colon tissue samples from the AOM/DSS model of colon cancer at 35 days. Mice from each group were euthanized at *day 35* and *day 70* as described in the methods. Colons were harvested and processed to determine the histology score at *day 35*. Values represent the mean ± SE. Representative H&E stained colon sections (100x magnification) are shown for each group. Arrows indicate areas of inflammation. Significant differences from the AOM + DSS only group are indicated by * (*p* < 0.05) and ** (*p* < 0.01).

To ensure that 2% DSS was effectively causing colitis in the treated mice, we determined the histology score of four euthanized mice (per group) at *day 35*. In Figure 1B, histology scores for ulceration and inflammation indicate that the AOM + DSS only group does have a significantly higher score than the AOM only group. As expected at this time point, despite the varying severity of colitis in the treatment groups, none of the mice had developed tumors.

**Figure 2:**
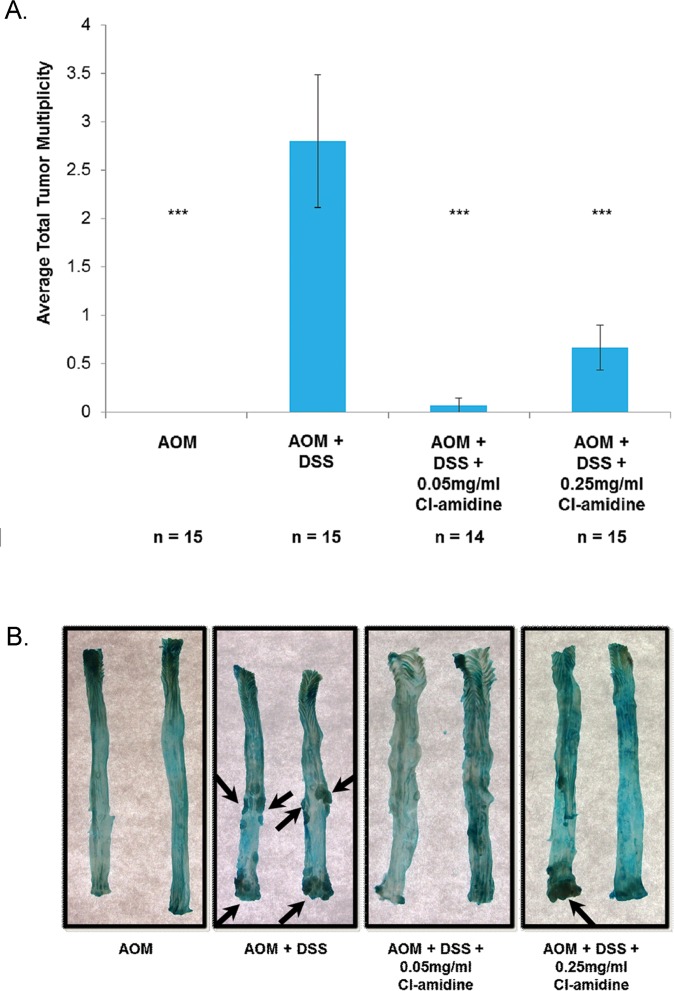
Tumor multiplicity is reduced in the colons of mice treated with Cl-amidine Mice from each group were euthanized at *day 70* as described in the methods. Colons were removed and cut longitudinally. **A.** Tumor multiplicity in the AOM/DSS model of colon cancer at *70 days*. Significant differences from the AOM + DSS only group are indicated by *** (*p* < 0.005). **B.** Representative methylene blue stained colons are shown for each group. Arrows indicate tumors.

At *day 70*, mice were euthanized, and we recorded the number mice with tumors (Table 1) and the average number of tumors per mouse in each group (Figure 2A; tumor multiplicity). We also measured the size of each tumor (Table 1) and captured representative images of the methylene blue stained colons (Figure 2B). No tumors were found in the control AOM only mice and, as predicted, the AOM + DSS only group (Group 2: 2.8 ± 0.68 tumors/animal) had the highest tumor multiplicity. Mice treated with Cl-amidine (Group 3: 0.07 ± 0.07 tumors/animal; Group 4: 0.67 ± 0.23 tumors/animal) had significantly reduced tumor multiplicity than the AOM + DSS only group. Interestingly, there was a significant difference in tumor multiplicity (*p* < 0.05) between the Cl-amidine treated groups where the higher dosage group (Group 4: 0.25 mg/mL Cl-amidine) had a higher tumor multiplicity than the lower dosage group (Group 3: 0.05 mg/mL Cl-amidine). Although at this point we are unable to explain this observation, perhaps a certain basal level of citrullination is necessary to carry out functions, such as apoptosis. Since the citrullination of proteins involved in apoptosis (e.g. vimentin, nucleophosmin, nuclear lamin C) facilitates the process of apoptosis [18-21], the higher dose of Cl-amidine may be inhibiting the citrullination necessary for the progression of apoptosis and aiding in the development of tumors, thus accounting for the increased tumor incidence. Nevertheless, the overall results from this model show for the first time that Cl-amidine is capable of preventing tumorigenesis associated with chronic colitis.

**Table 1:** Tumor incidence, multiplicity, and size are reduced in the colons of mice treated with Cl-amidine

	Tumor Incidence	Average # of tumors	Average tumor multiplicity <1mm	Average tumor multiplicity >1mm
AOM	0 (0%)	0	0 ± 0*	0 ± 0***
AOM + DSS	13 (86.7%)	2.80	0.67 ± 0.37	2.13 ± 0.53
AOM + DSS + 0.05mg/ml Cl-amidine	1 (7.1%)	0.07	0 ± 0*	0.07 ± 0.07***
AOM + DSS + 0.25mg/ml Cl-amidine	7 (46.7%)	0.67	0 ± 0*	0.67 ± 0.23*

Tumor incidence (the number of mice per group with tumors) is shown alongside the percentage of mice with tumors. The average number of tumors per mouse (tumor multiplicity) is shown and is also divided based on size (<1 mm and >1 mm at the largest dimension). Tumor multiplicity based on tumor size is given as mean ± standard error of the mean (SE). Significant differences from the AOM + DSS only group are indicated by * (*p*<0.05) and *** (*p*<0.005).

### Cl-amidine increases miR-16 expression and downregulates cell proliferative miR-16 targets in mice

**Figure 3:**
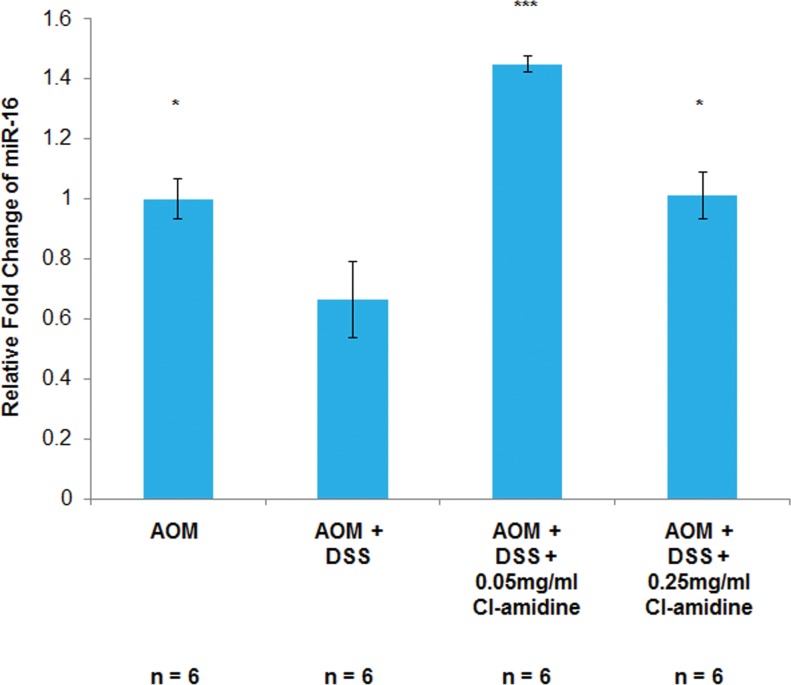
MiR-16 expression is increased in the colon epithelial cells of mice treated with Cl-amidine Mice from each group were euthanized at *day 35* and colons were removed to be processed to separate the colon inflammatory cells from the epithelial cells via magnetic microbeads. Then, as described in the methods, total RNA was extracted and primed to measure miR-16 expression using qPCR. MiR-16 expression across the treatment groups was quantified as the relative fold change as compared to AOM only. MiR-16 expression in colon epithelial (CD45-) cells at *day 35* in the AOM/DSS model of colon cancer is shown. Significant differences from the AOM + DSS only group are indicated by * (*p* < 0.05) and *** (*p* < 0.005).

We have previously shown that Cl-amidine increases miR-16 expression in a p53-dependent manner resulting in a cell cycle arrest *in vitro* [17]. Since the inhibition of cell proliferation is a goal of many anti-cancer drug therapies, we hypothesized that Cl-amidine is preventing tumorigenesis by increasing miR-16 expression *in vivo*. To test this, we measured miR-16 expression levels in isolated colon epithelial (CD45-) cells at *day 35* (see methods). We chose to investigate the miR-16 expression levels at the *day 35* time point because we were interested in the mechanism preventing tumorigenesis at *day 70*. Figure 3 shows the relative fold change in miR-16 levels in the epithelial cells. Consistent with our previously published *in vitro* data [17] and our current hypothesis, miR-16 expression in the AOM + DSS only group was significantly lower than the AOM only group and both Cl-amidine treated groups. Furthermore, the lower level of miR-16 expression in epithelial cells from the higher dosage group (0.25 mg/mL Cl-amidine), compared to the lower dosage group (0.05 mg/mL Cl-amidine), is highly suggestive of a direct correlation between the expression level of miR-16 and tumor incidence; however, the cause of this variability between Cl-amidine treatment groups is currently unknown.

**Figure 4:**
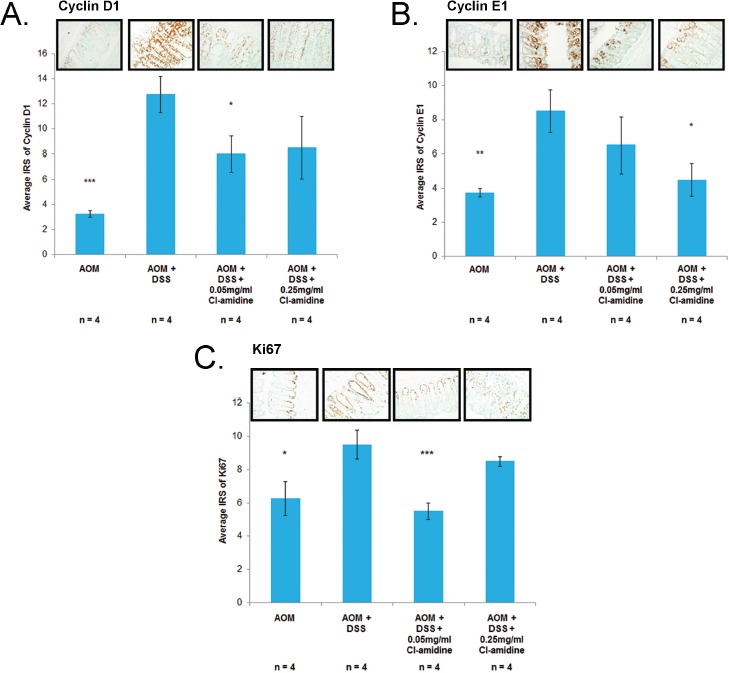
Cell proliferation proteins (Cyclin D1, Cyclin E1, and Ki67) are downregulated in mice treated with Cl-amidine Colons from 4 mice per group were euthanized at *day 35* and processed for IHC analysis. IRS of colons at *day 35* stained with **A.** Cyclin D1, **B.** Cyclin E1, both known targets of miR-16, and **C.** Ki-67, a cell proliferation marker. Representative sections of stained colons (400x total magnification) are shown. Significant differences from the AOM + DSS only group are indicated by * (*p* < 0.05), ** (*p* < 0.01), and *** (*p* < 0.005).

miR-16 has multiple cell proliferation targets, such as Cyclin D1 and Cyclin E1; supporting the premise that it is a tumor suppressor miRNA [17, 22-25]. If miR-16 expression is increased with Cl-amidine treatment (Figure 3), then we expect to see the downregulation of these cell proliferation targets of miR-16. Indeed, we confirmed that protein expression of Cyclins D1 and E1 was suppressed in the Cl-amidine treated groups when compared to the AOM + DSS only group (Figures 4A and 4B). To further verify the repression of cell proliferation in the mice treated with Cl-amidine, we performed IHC staining for the cell proliferation marker, Ki67, in colons collected at *day 35* (Figure 4C). In the colons collected at *day 35*, the IRS of the AOM + DSS only groups were significantly higher than the control AOM only groups (Figure 4A–4C). For Cyclin D1 and Ki-67 stained colon sections, the lower dose Cl-amidine group (0.05 mg/mL) had a lower IRS than the AOM + DSS only group; however, the group receiving 0.25 mg/mL Cl-amidine was not significantly lower. Interestingly, the IRS values of Cyclin D1 and Ki67 staining at *day 35* revealed a similar trend to the tumor incidence and miR-16 expression levels in colon epithelial cells. For Cyclin E1 stained colon sections, the groups treated with Cl-amidine both had lower IRS values than the AOM + DSS only group, but the lower dose Cl-amidine group (0.05 mg/mL) was not significantly less. These results are consistent with the hypothesis that Cl-amidine is suppressing tumorigenesis in our mouse model by inhibiting cell proliferation *via* increased miR-16 expression.

## DISCUSSION

**Figure 5:**
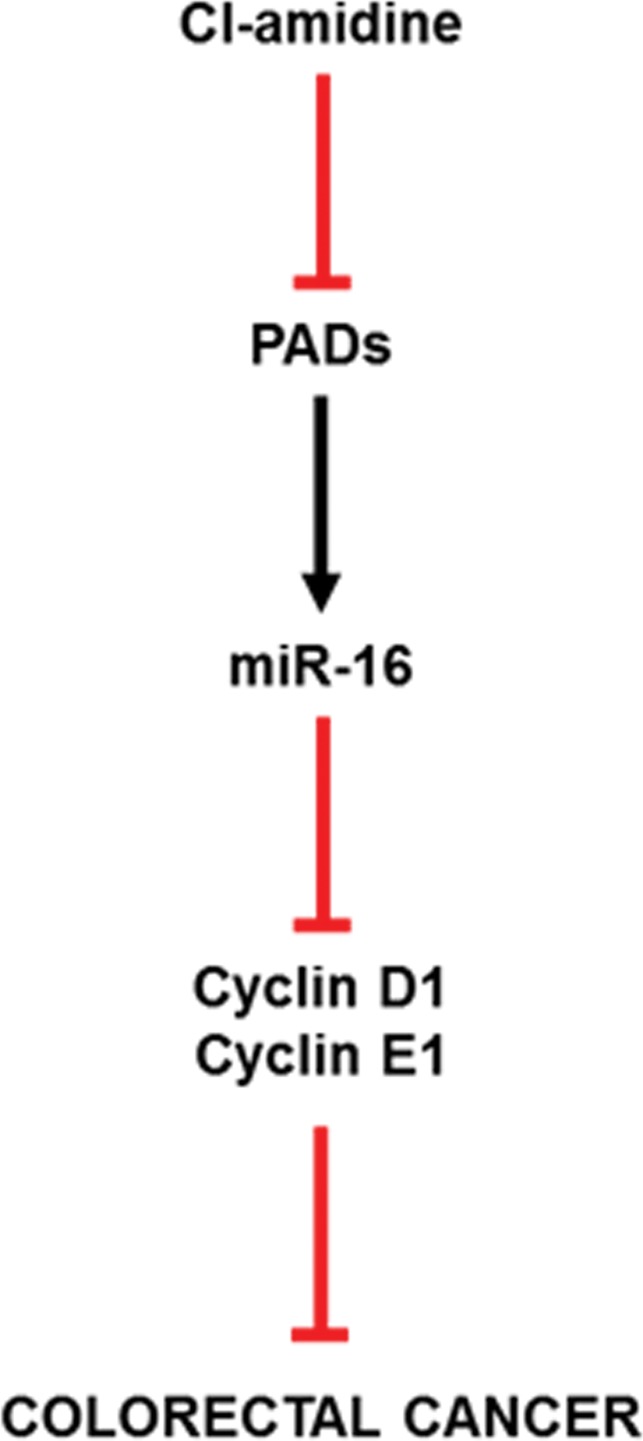
Proposed mechanism of action for Cl-amidine preventing CRC As evidenced in the results produced in this manuscript, Cl-amidine upregulates miR-16 expression in mice through PAD inhibition. At this point, the cell proliferation targets (Cyclin D1 and E1) of miR-16 are downregulated, accounting for the decreased tumorigenesis found at the endpoint of our study.

In this study, we provide evidence that Cl-amidine, a small molecule inhibitor of PADs, administered to mice in drinking water suppresses colitis (Figure 1) and tumorigenesis (Figure 2; Table 1). To uncover the mechanism by which Cl-amidine is acting, we revealed that Cl-amidine increases miR-16 expression in colon epithelial cells (Figure 3). Likewise, Cl-amidine treatment decreases protein expression of the miR-16 targets, Cyclins D1 and E1, and the cell proliferation marker, Ki67 (Figure 4). Figure 5 depicts the unique mechanism by which Cl-amidine prevents the tumorigenesis of CRC in our mouse model.

PADs are calcium-dependent. Therefore, they are usually inactive under physiological levels (10^−8^ to 10^−6^ M); and only activating during certain events (i.e. apoptosis and terminal epidermal differentiation) where calcium levels are above physiological concentrations [5, 18]. Interestingly, and important to this study, patients with active Crohn's disease or UC have moderately higher Ca^2+^ levels than healthy controls [26]. Not surprisingly, then, citrullination (the post-translationally modified product of active PADs) is elevated in colitis [27]. During apoptosis and terminal epidermal differentiation, PADs are found to citrullinate structural proteins, such as vimentin and filaggrin, causing partial unfolding of the proteins [5, 18]. The citrullination of these structural proteins promotes the overall degradation of the cells during cell death. PADs, specifically PAD4, have also been found to regulate gene expression [28-33]. Many of the genes that the PADs regulate are involved in the progression of the p53 pathway, including ING4, p300, and HDAC2 [30-32]. With the involvement of PADs in pathways that are crucial for proper cell growth and cell death, it is readily apparent that aberrant PAD activity can lead to deleterious consequences (e.g. abnormal DNA damage repair response, protein misfolding, protein inactivation).

Additionally, citrullination controls the expression of tumor suppressing miRNAs. miRNAs are regulators of genes that control various cell signaling processes, such as cell proliferation, apoptosis and stress response [34]. As a result of an inflammatory response, such as that of UC, miRNAs can be altered and expression levels can fluctuate [35]. Although other miRNAs have been found associated with either mouse or human UC, we focused here on miR-16 because our previous *in vitro* data showed that Cl-amidine induces miR-16 expression and decreases the expression of several miR-16 targets (i.e. Cyclins D1, D2, D3, E1, and CDK6) involved in the progression through the cell cycle [17]. Likewise, miR-16 is found at lower levels in CRC than in normal tissue [36]. One prospective pathway through which Cl-amidine may be upregulating miR-16 expression levels involves the tumor suppressor p53. p53 boosts the post-transcriptional maturation of miR-16 and Cl-amidine is found to increase miR-16 expression in a p53-dependent manner *in vitro* [17, 37-39].

In summary, we have shown that Cl-amidine suppresses colitis and, in this current study, suppresses tumorigenesis in mice given Cl-amidine dosages in drinking water [14]. Our current *in vivo* study is a substantial extension of our previous mechanistic *in vitro* data and suggests that increased miR-16 expression in mice treated with Cl-amidine results in decreased tumor formation [17]. When miR-16 is downregulated, this relieves its inhibition of cell proliferative targets, like Cyclin D1 and E1. Because our results demonstrate that Cl-amidine can suppress these targets of miR-16, this verifies the activity of miR-16 throughout our model. These findings display a significant development in our knowledge of *in vivo* mechanisms by which PAD inhibition can suppress colon cancer. Future studies will explore the mechanism(s) by which Cl-amidine is increasing miR-16 expression and will determine optimum dosages for preventing tumorigenesis. Overall, this study presents Cl-amidine as a viable cancer preventative therapy against colitis-associated colorectal cancer and provides an innovative mechanism of action involving the upregulation of miR-16, ultimately leading to decreased cell proliferation and prevention of tumorigenesis *in vivo*.

## MATERIALS AND METHODS

### Cl-amidine

The synthesis of Cl-amidine has previously been described [6, 40].

### AOM/DSS mouse model of colitis-associated colorectal cancer

This mouse model of colorectal cancer is outlined in Figure 1A. C57BL/6 male mice (8-12 weeks old; Jackson Laboratory) were used in accordance with protocols approved by the Institutional Animal Care and Use Committees of University of South Carolina. AOM (10 mg/kg) was injected into each mouse *via* intraperitoneal injection. The mice were divided into four groups where Group 1 received drinking water *ad libitum* throughout the experiment. One week after AOM injection, Groups 2-4 began cycles of 2% DSS (MP Biomedicals, Solon, OH; m.w. 36 000-50 000) given in the drinking water for 70 days. Groups 2-4 were subjected to three cycles, each consisting of one week with 2% DSS in the water followed by two weeks of normal drinking water. The DSS cycles were aimed to simulate the active inflammatory and remission states of UC. Groups 3 and 4 were administered 0.05 mg/mL and 0.25 mg/mL Cl-amidine, respectively, daily in the drinking water beginning at *day 14* and ending at *day 70*.

0.05 mg/mL and 0.25 mg/mL, which is the equivalent of 10 mg/kg/day and 50 mg/kg/day per mouse, respectively; or the human daily equivalent of 48.6 mg and 243 mg, respectively. Calculations are as follows: assuming an average mouse weighs 25 g and drinks approximately 5 mL per day, then 0.25 mg / 25 g x 1000 g / 1 kg = 10 mg/kg daily. The human equivalent dose (HED, mg/kg) = animal dose (mg/kg) x [Animal Km / Human Km] [41]. The HED (mg/kg) for mouse = 10 mg/kg x [3/37] = 0.81 mg/kg. Therefore, assuming an adult human weighs approximately 60 kg on average, the human equivalent is 0.81 mg/kg x 60 kg = 48.6 mg daily. Using the same equations, the 0.25 mg/mL mouse dosage is approximately the equivalent of 243 mg daily for humans. We chose these doses because our previous work has shown that 5 - 75 mg/kg/mouse per day was effective in a dose-responsive manner at suppressing DSS-induced colitis in mice [14]. Since 5 mg/kg/day was only modestly effective, and 75 mg/kg/day is the Maximal Tolerable Dose, we wanted to investigate the potency of multiple doses within this range [14, 15]. Thus we chose 0.05 mg/mL and 0.25 mg/mL delivered in the drinking water. We reasoned Cl-amidine would work better delivered in the drinking water because Cl-amidine has a short half-life *in vivo* [15]. Therefore delivering the same amount throughout the day over a long period of time (in water) *versus* a bolus amount (oral gavage) would be a better method with short-life compounds. We also determined that Cl-amidine is stable in DSS-spiked water for 48 h (data not shown) and the mice drink approximately the same amount of water daily regardless of treatment (Supplementary Figure S1).

At *day 35*, after 1.5 cycles of DSS, 10 mice from each group (1-4) were euthanized to ensure that the DSS was effective and for further analysis. Colons from each mouse were removed, cut longitudinally, and washed with 1x phosphate buffered saline (PBS). The colons from four mice from each group were Swiss-rolled, fixed in 10% buffered formalin overnight, paraffin embedded, and then sectioned for histopathology/immunohistochemistry. The colons from the remaining six mice/group were incubated in 10% fetal calf serum/5 mM ethylenediaminetetraacetic acid (EDTA)/Ca^2+^/Mg^2+^ free PBS for 15 min. Colons were then shaken gently for 10 sec and the single cell suspension, consisting of epithelial and inflammatory cells, collected in the supernatant. Inflammatory cells were separated from epithelial cells using CD45+ magnetic microbeads that select for the inflammatory cells, according to kit instructions (mouse CD45^+^ MicroBeads, Miltenyi Biotec, Auburn, CA). These separated cells were immediately processed for miRNA analysis to avoid any breakdown of the RNA integrity.

At *day 70*, the remaining 15 mice from each of the groups were euthanized. Colons were removed, cut longitudinally, and washed with 1x PBS. Tumor incidence and size were recorded for each colon. Colons from each group were processed for histopathology/immunohistochemistry in the same manner as the colons harvested at *day 35*.

### Quantification of inflammation

Colons prepared for histopathology were stained with hematoxylin and eosin (H&E). Sectioned H&E stained samples were examined microscopically and scored as previously described [14]. Briefly, the histology score for a sample was determined by adding the scores for inflammation severity, inflammation extent, and crypt damage; then, multiplying by the score for percent area involvement. The inflammation severity was scored on a scale of 0-3: 0 (no inflammation), 1 (minimal), 2 (moderate), and 3 (severe). Inflammation extent was scored on a scale of 0-3: 0 (no inflammation), 1 (mucosa only), 2 (mucosa and submucosa), and 3 (transmural). Crypt damage was scored on a scale of 0-4: 0 (no crypt damage), 1 (one-third of crypt damaged), 2 (two-thirds damaged), 3 (crypts lost and surface epithelium intact), and 4 (crypts lost and surface epithelium lost). Percent area involvement was scored on a scale of 0-4: 0 (0% involvement), 1 (1-25%), 2 (26-50%), 3 (51-75%), and 4 (76-100%). The minimum possible score is 0 and the maximum possible score is 40.

### MiR-16 expression

Total miRNA was extracted from separated inflammatory (CD45+) and epithelial (CD45-) cells according to miRNeasy Mini Kit instructions (Qiagen). RNA concentration and stability were measured by the Nanodrop 2000 (Nanodrop, Wilmington, DE). 10 ng of total RNA were used to make cDNA using the TaqMan MicroRNA Reverse Transcription kit (Applied Biosystems, Foster City, CA) according to kit instructions. We also used hsa-miRNA-16 primers for miR-16 detection and small nuclear protein RNU6B (U6) for normalization (Applied Biosystems, Foster City, CA). Quantitative Real-Time PCR to measure miR-16 and U6 was performed using the TaqMan miRNA Assay (Applied Biosystems, Foster City, CA) with the 7300 PCR Assay System (Applied Biosystems, Foster City, CA). The relative fold change (FC) of miR-16 expression, as compared to U6 expression, was determined based on the comparative threshold cycles (Ct) of miR-16 and U6. All samples were analyzed in triplicate.

### Immunohistochemical staining

Paraffin-embedded sections of mouse colonic tissue were incubated (4°C) in Cyclin D1 (Rabbit monoclonal, cat# TA307019, diluted 1:100, Origene, Rockville, MD), Cyclin E1 (rabbit polyclonal, cat# TA311853, diluted 1:1000, Origene, Rockville, MD), and anti-Ki67 (Rabbit polyclonal, cat# PA5-19462, diluted 1:5000, Pierce, Rockford, IL) primary antibodies by slow rocking overnight with Antibody Amplifier (ProHisto, Columbia, SC) to ensure even and reproducible staining. Samples were then processed using the EnVision+ System HRP kits (DAKO, Carpinteria, CA) according to kit instructions. The chromagen, diaminobenzidine, was added and then the samples were counterstained with methyl green. Stained tissues were objectively scored based on two criteria: 1) percentage of tissue stained, and 2) the staining intensity. The percentage of tissue stained was scored on a scale of 0-5: 0 (0% positive staining), 1 ( < 10%), 2 (11-25%), 3 (26%-50%), 4 (51%-80%), or 5 ( > 80%). Staining intensity was scored on a scale of 0-3: 0 (Negative staining), 1 (Weak), 2 (Moderate), or 3 (Strong). Scores from each criteria were multiplied to get the final immunoreactivity score (IRS).

### Statistical analysis

Mean differences between groups were compared by one-way ANOVA with Scheffe multiple comparison tests. The *p*-value chosen for significance in this study was 0.05.

## SUPPLEMENTARY MATERIAL FIGURE


