# Supplementary material for: Molecular targeting of protein arginine deiminases to suppress colitis and prevent colon cancer
Source: Oncotarget. 2015 Sep 30;6(34):36053–62. doi: 10.18632/oncotarget.5937 (PMC4742161; doi:10.18632/oncotarget.5937)
Supplement: Supplementary file 1 [file oncotarget-06-36053-s001.pdf]

Molecular targeting of protein arginine deiminases to suppress colitis and prevent colon cancer

Supplementary Material

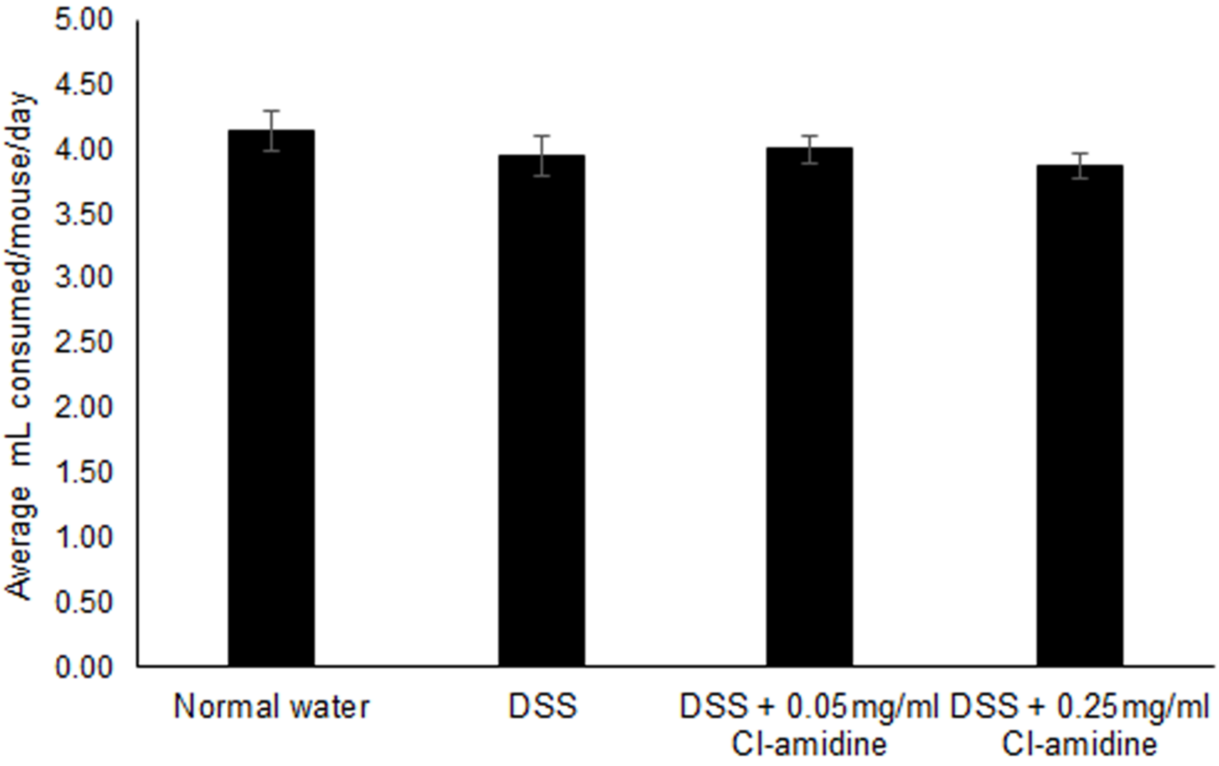

Supplementary Figure 1: Average water intake for mice given 2% DSS and Cl-amidine treatments. Mice were given indicated treatments in water to represent treatments used in the in vivo model of colitis-associated colorectal cancer. Daily water consumption was measured for 7 days (Normal water and DSS only groups) and 10 days (DSS + Cl-amidine groups). No significant differences were reported between the groups measured.
